# Factors Affecting Knowledge of Attention-Deficit Hyperactivity Disorder Among Female Primary Schoolteachers in Taif City, Saudi Arabia

**DOI:** 10.7759/cureus.40057

**Published:** 2023-06-06

**Authors:** Razan H Alzahrani, Nesrin K Abd El-Fatah

**Affiliations:** 1 Preventive Medicine, Ministry of Health, Taif, SAU; 2 Preventive Medicine, Alexandria University, Alexandria, EGY

**Keywords:** female teachers, saudi arabia, primary schools, misconceptions, knowledge, attitude, attention-deficit/hyperactivity disorder

## Abstract

Background

Attention-deficit hyperactivity disorder (ADHD) is a public health challenge because it may contribute to academic and psychological difficulties among school children. Though ADHD is a common problem, Taif teachers' knowledge of the disease has never been assessed. Therefore, this study aimed to determine factors that influence ADHD knowledge among female primary schoolteachers in Taif City, Saudi Arabia.

Methods

This cross-sectional study was conducted on 359 female schoolteachers recruited by stratified random sampling. Participants self-reported demographic and personal data and completed the validated Arabic version of the knowledge of ADHD scale and teacher’s attitude toward ADHD questionnaires.

Results

In Taif, it was determined that 96.4% of female primary schoolteachers had insufficient knowledge of ADHD, notably with nature, causes, consequences, and treatment knowledge. In contrast, 40% had adequate knowledge of the condition's symptoms and diagnosis, and 97.5% exhibited a favorable attitude. There is significantly higher knowledge among private schoolteachers, those who freshly graduated, specialize in learning difficulties, who attended any course/training about ADHD, and whoever taught ADHD children. There was a significant positive weak correlation between teachers’ knowledge of ADHD and their attitude. Regression analysis revealed that female schoolteachers specialized in learning difficulties show significantly higher knowledge scores, teachers who never taught an ADHD child had a reduction of 94.6 % in ADHD knowledge, and any increase in the number of ADHD children teachers teaches has significantly increased teachers’ knowledge (Overall Model: Chi-Square X2= 69.514, p < 0.000**).

Conclusions

Our study showed that there was a serious knowledge gap on ADHD among Taif female primary schoolteachers. Therefore, it is strongly recommended to boost teachers' knowledge of ADHD, especially at government schools, through conducting training courses, distributing leaflets intended to address ADHD, and launching awareness campaigns through the media, including social media, television, and radio. It is also recommended that education faculty curricula should include more information on ADHD.

## Introduction

Attention-deficit hyperactivity disorder (ADHD) is a critical disorder affecting school-aged children [1]. It includes inattention (not being able to keep focus), hyperactivity (excess movement that is not fitting to the setting), and impulsivity (hasty acts that occur at the moment without thought). The latest global prevalence of ADHD was 7.6% [2]. Children with ADHD are at risk for academic and behavioral problems [1]. The prevalence of ADHD in Arab countries among schoolchildren aged 6-12 years ranges between 7.8% and 11.1%, while it was higher, at 16%, in the studies that included younger children aged between 3 and 15 years of age [3]. A recently published meta-analysis of a total of 33 studies from the six Gulf Cooperation Council countries found the prevalence of ADHD as per the Vanderbilt ADHD Diagnostic Rating Scale, Attention-Deficit Disorders Evaluation Scale, and the Strengths and Difficulties Questionnaire to be 13.12%, 26.13%, and 12.83%, respectively [4]. In Saudi Arabia, the prevalence of ADHD was 2.7% in children aged 7-9 years in the southern region [5] and 3.5% in girls aged 6-15 years in the eastern region [3], and another two studies have reported the 11.6% and 16.3% prevalence of ADHD among primary school students [6,7]. However, a study done at primary schools in Dammam reported a higher prevalence of the inattention group as compared to hyperactivity among male students [8].

Studies of ADHD-associated factors focus mainly on prenatal and perinatal factors (e.g., maternal alcohol consumption and smoking, maternal stress, low birth weight, and prematurity), environmental lead exposure [9], and traumatic brain injuries [10]. In addition, high maternal and paternal education levels were associated with a decreased risk for ADHD [11]. The signs and symptoms of ADHD can be categorized into two types of behavioral problems: inattentiveness and hyperactivity-impulsiveness. In young children, the two types of behavioral problems associated with ADHD coexist. The main signs and symptoms of inattentiveness form include difficulty in maintaining attention and organizing tasks, failure to pay close attention to details, inability to listen, being easily distracted, appearing forgetful, and losing objects. Meanwhile, the hyperactivity and impulsivity types struggle to remain seated when it’s required, exhibit excessive fidgeting and talking, experience feelings of restlessness, struggle to play quietly or wait for turns, and interrupt or intrude on others [12]. The best available evidence for ADHD diagnosis and most frequently used is the Diagnostic and Statistical Manual of Mental Disorders-Fifth Edition (DSM-5) classification criteria. It defines four dimensions of ADHD: ADHD primarily of the inattentive presentation (ADHD/I), ADHD of the hyperactive-impulsive presentation (ADHD/HI), combined presentation, and other specified and unspecified ADHD. However, in most cases, the use of neuropsychological testing has not been found to improve diagnostic accuracy [13]. An 11-year follow-up study of 140 patients diagnosed with ADHD at ages 6-18 found that ADHD persists into adulthood and 35% had syndromic persistence, 22% had symptomatic persistence, and 15% had functional persistence [14].

The American Academy of Pediatrics recommends a combination of medication and behavior therapy, parent training on behavior management, behavioral classroom intervention, educational interventions, and individualized instructional supports, including school environment, class placement, instructional placement, and behavioral supports, which are a necessary part of any treatment plan for ADHD children aged 6 years to 12 years. Some nonmedication treatments for ADHD-related problems have either too little evidence to recommend them or have been found to have little or no benefit. These include mindfulness, cognitive training, diet modification, electroencephalography biofeedback, and supportive counseling [13]. However, nutritional management is an important aspect that has been neglected; food additives, refined sugars, food sensitivities/allergies, and fatty acid deficiencies are linked to ADHD, and they can negatively impact their behavior [15].

Since ADHA impairs academic performance, which is culturally highly valued in the Arab world [1], teachers have an essential role in reporting ADHD at a higher frequency than parents [5]. Many studies revealed a significant knowledge gap in different aspects of ADHD among schoolteachers, which impacts their attitudes. The teachers’ knowledge and perception of ADHD were mostly acquired from television, magazines, and books [16]. The lack of knowledge of ADHD among teachers was apparent and of different rates among different studies worldwide. The scores of teachers’ good knowledge ranged from 46% to 66% in Texas (2012) [17], 17.9% to 46.2% in Egypt (2016) [16], and only 19.4% in Thailand (2019) [18]. The female schoolteachers in Makkah also reported poor knowledge of ADHD (58.4%) [19].

ADHD tends to be noticed at an early age and may become more noticeable when a child starts school. Most cases are diagnosed when children are 6 to 12 years old [3,5]. Given the potential that teachers play an essential role in the discovery of ADHD among children as children spend most of their time in the classroom and know how ordinary students typically behave, exploring teachers’ ADHD knowledge status and their associations is crucial so that preventive measures could be contemplated. To date, research on teachers’ knowledge and attitude toward ADHD in primary schools in Saudi Arabia is still limited. Accordingly, this study aimed to determine the knowledge and attitude toward ADHD among female primary schoolteachers in Taif City, Saudi Arabia, as well as their association with their demographic characteristics so that differentiated educational intervention can be planned and implemented for teachers about ADHD.

## Materials and methods

Design and study population

A cross-sectional study was carried out on Saudi female primary schoolteachers from the private and governmental sectors in Taif City, Saudi Arabia, between December 2022 to April 2023. The study was conducted at primary schoolteachers, under the Ministry of Education in Taif City in the western province of Saudi Arabia. The total number of girls’ primary schools in Taif City is 112. A multi-stage stratified random sampling technique was used. In the first stage of sampling, Taif City was divided into four educational districts. In the second stage, stratification was based on the type of school; six primary schools (three private and three governmental) were randomly selected from each district. In the subsequent stage, the predetermined sample was proportionally allocated to the selected schools according to their teachers’ numbers. Finally, a systematic random sampling of every second female teacher was used to choose participants from each school until the predetermined sample was fulfilled. The study authors distributed the study questionnaire themselves. In each district, questionnaires were to be gathered over the course of a month from different schools. Teachers who are non-Saudi or those working in administrative positions like principals and admin staff of the schools were excluded. It is noteworthy to mention that the response rate was 96.24%. Using the 2002 Epi Info™ software (Centers for Disease Control and Prevention, Atlanta, Georgia), a minimum required sample of 373 samples was determined based on the knowledge of teachers about ADHD (41.61%) [20] with a precision of 5%, a confidence level of 95%, and an error of 0.05. The research received institutional ethical approval (IRB registration number: HAP-02-T-067), and permission from the Ministry of Education to visit schools was obtained. A written consent was taken from all the participants before they answered the questions and confidentiality was assured. The research was conducted in accordance with the Declaration of Helsinki.

Measures

Female teachers filled out a self-reported questionnaire that consisted of three sections:

Demographic Questionnaire

Section A comprised demographic questions that covered age, nationality, marital status, educational level, occupational experience years, school type, teaching specialty, and previous experience with ADHD students. In addition, this study measured where the participants received information (books, Internet websites, social media, friends, training, etc.) about ADHD or whether they already have a child with ADHD.

Knowledge of Attention‑Deficit Disorders Scale

Section B of the tool assessed the schoolteacher’s knowledge of ADHD by using the Knowledge of Attention-Deficit Disorders Scale (KADDS). It is a widely used screening instrument used to assess the level of knowledge of teachers regarding ADHD and was developed by do Sciutto and his colleagues. It has been established as a reliable and valid instrument (Cronbach’s α = 0.71 for each sub-scale and 0.86 for the scale as a whole) [21]. The internal consistency of the Arabic version of this scale and Cronbach’s alpha value, in the previous study, was 0.76 which indicates adequate internal consistency [22]. KADDS comprises 36 questions each with three response options, varying from three option response formats: true (1), false (2), or don’t know (DK) (3). The scale contains three subscales: symptoms/diagnosis of ADHD (9 items), the treatment of ADHD (12 items), and general knowledge of the nature, causes, and outcome of ADHD (15 items). It also includes three additional questions (not in the original KADDS) not classified into any of the subscales. One point was given for correct answers and 0 for incorrect ones, and the lack of knowledge of the teachers and item responses were summed. Thus, the possible scores ranged from 0 to 36. Higher scores indicate a higher level of knowledge.

Teachers Attitudes Toward ADHD

Section C of the study instrument assessed the teacher’s attitudes toward ADHD by using a 12-item. The questionnaire was already available in the Arabic language, and it showed adequate reliability among Saudi teachers as evaluated by Cronbach’s alpha (α= 0.84) to assess teachers' attitudes toward ADHD [23]. Items were assessed by using a five-point scale ranging from strongly disagree (1) to strongly agree (5). Finally, item responses were summed, and their score ranged from 12 to 60 points. Lower scores indicate lower teacher’s attitudes toward ADHD and higher scores indicate higher attitudes.

Statistical analysis

Statistical analyses were performed using SPSS Statistics version 24 (IBM Corp. Released 2016. IBM SPSS Statistics for Windows, Version 24.0. Armonk, NY: IBM Corp.). The teacher’s knowledge score was computed, converted into a percentage score, and categorized according to the total knowledge score into two categories as described in a previous study: insufficient (mean score ≤60%) and good (mean score > 60%). Likewise, the schoolteacher’s attitude score was computed and converted into a percentage score, and teachers who scored ≥ 60.0% were considered as having a favorable attitude toward ADHD [23,24]. Descriptive statistics, including frequencies and percentages, were used for categorical variables; median and range were used for continuous variables after determining the normality using the Shapiro-Wilk test. The Shapiro-Wilk statistic test showed that knowledge and attitude scores were not normally distributed. The overall reliability of the Arabic version of KADDS was assessed in which Cronbach’s α = 0.94. Meanwhile, the teacher’s attitudes toward ADHD questionnaire demonstrated good reliability Cronbach’s alpha = 0.81. The prevalence of insufficient knowledge and unfavorable attitude toward ADHD was computed and reported as percentages. Spearman Rho correlation coefficients were determined to test the association between the knowledge subscales and attitude scores. Kruskal-Wallis test (multiple groups) and Mann-Whitney U test (dichotomous variables) were used to compare the teachers’ knowledge and attitude toward ADHD between the different demographic subgroups, teaching specialty, history of attending any course/training about ADHD, assessing a child suspected to have ADHD or teaching a child who has ADHD, and main sources of information about ADHD. Finally, a binary logistic regression model for knowledge (dummy variable) was conducted to determine factors that have a statistically significant association. For all statistical tests, a significance level was determined below 5% and quoted as two-tailed hypothesis tests.

## Results

The characteristics of the study participants are presented in Table 1. A total of 359 female teachers aged between 24 and 60 years completed the survey. More than three-quarters of the studied teachers were married and had a bachelor’s degree, while less than 4% (3.6% n=13) were holding postgraduate degrees. The majority of the teachers worked at governmental schools (79.1%, n=284), taught more than one school grade, and were special education teachers (96.7%, n=347) (language, science, learning difficulties, etc.). Additionally, 15% of the teachers had more than 26 years of experience, while more than 40% of the teachers had 6 to 15 years. The median and IQR for years of teaching experience were 13 and 1-37, respectively. The majority had heard about ADHD before, and their primary sources of information were social media (35.2%, n=119) and websites and scientific articles (30.8%, n=104). Only 16% of female schoolteachers at Taif's primary schools have received training courses about ADHD, and almost half of the participants (49%) have had varying degrees of experience working with children who have the disorder (Table 1).

**Table 1:** Demographic characteristics and source of information regarding knowledge and attitude toward ADHD among female primary schoolteachers in Taif City, Saudi Arabia (N =359) # multiple responses ADHD: attention-deficit hyperactivity disorder

	Categories	Frequency	Percent
Age group (years)	24-33	77	21.4 %
	34-43	139	38.7 %
	44-53	125	34.8 %
	54-63	18	5 %
Marital status	Single	48	13.4%
	Married	275	76.6%
	Divorced	28	7.8%
	Widow	8	2.2%
Level of education	High school or less	68	18.9%
	Bachelor’s degree	278	77.4%
	Postgraduate degree	13	3.6%
School type	Governmental school	284	79.1%
	Private school	75	20.9%
Teaching grade #	1^st^ grade	144	40.1%
	2^nd^ grade	131	36.5%
	3^rd^ grade	137	38.2%
	4^th^ grade	156	43.5%
	5^th^ grade	140	39.0%
	6^th^ grade	150	41.8%
Teaching specialty #	Languages	84	23.4%
	Mathematics	45	12.5%
	Science	32	8.9%
	Education	79	22.0%
	Kindergarten	13	3.6%
	Learning difficulties	18	5%
	General	12	3.3%
	Digital education	13	3.6%
	Physical education	13	3.6%
	Life skills	50	13.9%
Years of teaching experience	Equal or less than 5	73	20.3%
	6-15	148	41.2%
	16-25	82	22.8%
	Equal or more than 26	56	15.6%
Have you ever heard about ADHD?	No	21	5.8%
	Yes	338	94.2%
Main source of information about ADHD (n=338)	Specialized books and magazines	34	9.5%
	Websites and scientific articles	104	29%
	Television/media	62	17.3%
	Social media	119	33.1V
	Scientific courses	4	1.1%
	During the study years	3	0.8%
	During the work	4	1.1%
	I have an ADHD child	8	2.2%
Have you ever attended any courses/training about ADHD?	No	301	83.8%
	Yes	58	16.2%
During the years of teaching, do you think you have enough information about ADHD?	No	152	42.3%
	Yes	58	16.2%
	Not sure	149	41.5%
Have you ever been asked to assess a child suspected to have ADHD?	No	284	69.1%
	Yes	111	30.9%
Have you ever taught a child who has ADHD?	No	183	51%
	Yes	176	49%
Number of children with ADHD you taught in years (n=176)	1	53	14.8%
	2	56	15.6%
	3	21	5.8%
	Equal or more than 4	46	12.8%

**Figure 1:**
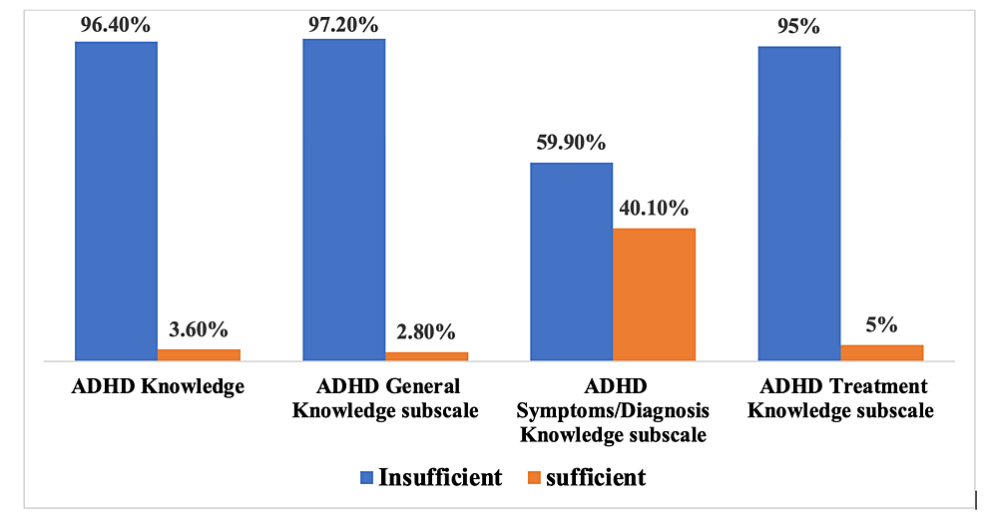
Percentage of schoolteachers with sufficient knowledge regarding ADHD and its three subscales ADHD: attention-deficit hyperactivity disorder

ADHD knowledge was classified into a sufficient knowledge group (3.6%) and an insufficient knowledge group (96.4%). The median ADHD knowledge score was 13 and IQR was 0-25. Different rates of ADHD knowledge subscales are presented in Figure 1.

Table 2 presents teachers’ responses on the general ADHD knowledge subscale. The lowest proportion of correct responses (1.7%) were on items 30 (“In very young children (less than 4 years old), the problem behaviors of ADHD children (e.g., hyperactivity, inattention) are distinctly different from age-appropriate behaviors of non-ADHD children.”) and 24 ("A diagnosis of ADHD by itself makes a child eligible for placement in special education. "). The majority of teachers (61.6%) selected the “don’t know” option on item 6 (“ADHD is more common in the 1st degree biological relatives (i.e., mother, father) of children with ADHD than in the general population.”). Likewise, the majority of teachers (55.2%) selected the “don’t know” option on item 11 (“It is common for ADHD children to have an inflated sense of self-esteem or grandiosity.”). For the symptoms/diagnosis subscale, the highest incorrect responses (44.3%) were on item 7. Regarding teachers’ responses on the ADHD treatment subscale, the highest proportion of correct responses (79.9%), was on item 10 (“Parent and teacher training in managing an ADHD child is generally effective when combined with medication treatment.”), while the highest proportion of incorrect responses (70.2%) was on item 23 (“Reducing dietary intake of sugar or food additives is generally effective in reducing the symptoms of ADHD.”). The highest percentage of "don’t know" responses (71.3%) on items 8 and 35 were pointing to a lack of treatment knowledge among the majority of the teachers (Table 2).

**Table 2:** Knowledge about ADHD among female primary schoolteachers in Taif City (n=359) ADHD: attention-deficit hyperactivity disorder

	Correct answer	True number (%)	False number (%)	Don’t know
General information knowledge subscale				
Most estimates suggest that ADHD occurs in approximately 15% of school-age children.	False	100 (27.9%)	50 (13.9%)	209 (58.2%)
ADHD children are typically more compliant with their fathers than with their mothers.	True	76 (21.2%)	78 (21.7%)	205 (57.1%)
ADHD is more common in the first degree of biological relatives (i.e., mother, father) of children with ADHD than in the general population.	True	88 (24.5%)	50 (13.9%)	221 (61.6%)
It is possible for an adult to be diagnosed with ADHD.	True	148 (41.2%)	42 (11.7%)	169 (47.1%)
Symptoms of depression are found more frequently in ADHD children than in non-ADHD children.	True	133 (37%)	31 (8.6%)	195 (54.3%)
Most ADHD children "outgrow" their symptoms by the onset of puberty and subsequently function normally in adulthood.	False	140 (39%)	31 (8.6%)	188 (52.4%)
If an ADHD child is able to demonstrate sustained attention to video games or TV for over an hour, that child is also able to sustain attention for at least an hour of class or homework.	False	145 (40.4%)	76 (21.2%)	138 (38.4%)
A diagnosis of ADHD by itself makes a child eligible for placement in special education.	False	229 (63.8%)	16 (4.5%)	114 (31.8%)
ADHD children generally experience more problems in novel situations than in familiar situations.	False	195 (54.3%)	14 (3.9%)	150 (41.8%)
There are specific physical features that can be identified by medical doctors (e.g., pediatricians) in making a definitive diagnosis of ADHD.	False	142 (39.6%)	41 (11.4%)	176 (49%)
In school-age children, the prevalence of ADHD in males and females is equivalent.	False	87 (24.2%)	87 (24.2%)	185 (51.5%)
In very young children (less than four years old), the problem behaviors of ADHD children (e.g., hyperactivity, inattention) are distinctly different from age-appropriate behaviors of non-ADHD children.	False	184 (51.3%)	6 (1.7%)	169 (47.1%)
Children with ADHD are more distinguishable from normal children in a classroom setting than in a free-play situation.	True	242 (67.4%)	23 (6.4%)	94 (26.2%)
The majority of ADHD children evidence some degree of poor school performance in the elementary school years.	True	210 (58.5%)	42 (11.7%)	107 (29.8%)
Symptoms of ADHD are often seen in non-ADHD children who come from inadequate and chaotic home environments.	True	142 (39.6%)	51 (14.2%)	166 (46.2%)
Symptoms/diagnosis of ADHD knowledge subscale				
ADHD children are frequently distracted by extraneous stimuli.	True	287 (79.9%)	13 (3.6%)	59 (16.4%)
In order to be diagnosed with ADHD, the child's symptoms must have been present before age 7.	True	167 (46.5%)	38 (10.6%)	154 (42.9%)
One symptom of ADHD children is that they have been physically cruel to other people.	False	159 (44.3%)	77 (21.4%)	123 (34.3%)
ADHD children often fidget or squirm in their seats.	True	293 (81.6%)	8 (2.2%)	58 (16.2%)
It is common for ADHD children to have an inflated sense of self-esteem or grandiosity.	False	103 (28.7%)	58 (16.2%)	198 (55.2%)
ADHD children often have a history of stealing or destroying other people's things.	False	107 (29.8%)	78 (21.7%)	174 (48.5%)
Current wisdom about ADHD suggests two clusters of symptoms: One of inattention and another consisting of hyperactivity/impulsivity.	True	196 (54.6%)	10 (2.8%)	153 (42.6%)
In order to be diagnosed with ADHD, a child must exhibit relevant symptoms in two or more settings (e.g., home, school).	True	229 (63.8%)	11 (3.1%)	119 (33.1%)
ADHD children often have difficulties organizing tasks and activities.	True	240 (66.9%)	24 (6.7%)	95 (26.5%)
ADHD treatment knowledge subscale				
Current research suggests that ADHD is largely the result of ineffective parenting skills.	False	68 (18.9%)	168 (46.8)	123 (34.3%)
Antidepressant drugs have been effective in reducing symptoms for many ADHD children.	True	73 (20.3%)	30 (8.4%)	256 (71.3%)
Parent and teacher training in managing an ADHD child is generally effective when combined with medication treatment.	True	287 (79.9%)	1 (0.3%)	71 (19.8%)
When treatment of an ADHD child is terminated, it is rare for the child's symptoms to return.	False	58 (16.2%)	90 (25.1%)	211 (58.8%)
Side effects of stimulant drugs used for the treatment of ADHD may include mild insomnia and appetite reduction.	True	97 (27%)	21 (5.8%)	241 (67.1%)
Individual psychotherapy is usually sufficient for the treatment of most ADHD children.	False	98 (27.3%)	86 (24%)	175 (48.7)
In severe cases of ADHD, medication is often used before other behavior modification techniques are attempted.	True	139 (38.7%)	33 (9.2%)	187 (52.1%)
Reducing dietary intake of sugar or food additives is generally effective in reducing the symptoms of ADHD.	False	252 (70.2%)	11 (3.1%)	96 (26.7%)
Stimulant drugs are the most common type of drug used to treat children with ADHD	True	62 (17.3%)	68 (18.9%)	229 (63.8%)
Behavioral/psychological interventions for children with ADHD focus primarily on the child's problems with inattention.	False	157 (43.7%)	25 (7%)	177 (49.3%)
Electroconvulsive therapy (i.e., shock treatment) has been found to be an effective treatment for severe cases of ADHD.	False	49 (13.6%)	54 (15%)	256 (71.3%)
Treatments for ADHD which focus primarily on punishment have been found to be the most effective in reducing the symptoms of ADHD.	False	41 (11.4%)	189 (52.6%)	129 (35.9%)

**Figure 2:**
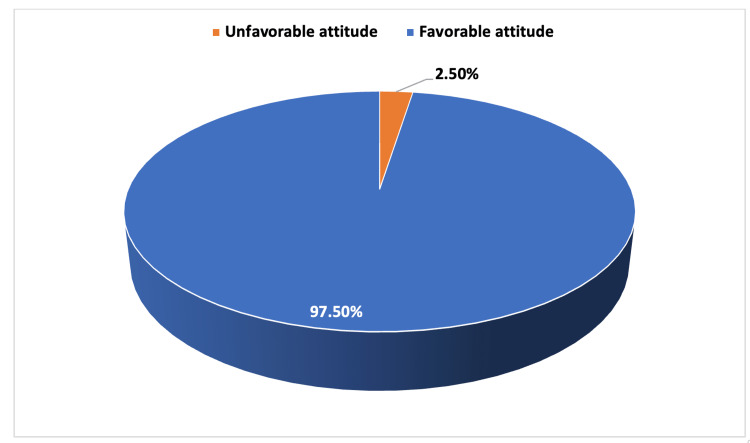
Percentage of schoolteachers with a favorable attitude toward ADHD

The attitude toward ADHD among female primary schoolteachers in Taif City is illustrated in Figure 2 and Table 3. The majority (97.5%) showed a favorable level of attitude toward ADHD as shown in (Figure 2). The median and IQR for attitude toward ADHD were 48 and 12-60, respectively. More than 50% of schoolteachers (57.7%, n=207) strongly agree that teacher training is important in the behavioral management of ADHD and treatment should be done if recommended by doctors. Items related to the causality of ADHD were more marked as neutral by the teachers, i.e., they thought that children develop ADHD because they want attention.

**Table 3:** Attitude toward ADHD among female primary schoolteachers in Taif City (n=359) ADHD: attention-deficit hyperactivity disorder

	Strongly disagree	Disagree	Neutral	Agree	Strongly agree
Special teaching techniques are helpful in managing ADHD.	3 (0.8%)	7 (1.9%)	38 (10.6%)	141 (39.3%)	170 (47.4%)
Behavior management is an effective treatment for ADHD.	3 (0.8%)	4 (1.1%)	37 (10.3%)	149 (41.5%)	166 (46.2%)
Training teachers in behavior management is important for ADHD.	4 (1.1%)	6 (1.7%)	25 (7%)	117 (32.6%)	207 (57.7%)
ADHD results from parents being inconsistent with rules and consequences.	8 (2.2%)	31 (8.6%)	84 (23.4%)	147 (40.9%)	89 (24.8%)
Some children develop ADHD because they want attention.	10 (2.8%)	66 (8.4%)	95 (26.5%)	130 (36.2%)	58 (16.2%)
Improving the parenting skills of parents of children with ADHD would benefit their child.	1 (0.3%)	10 (2.8%)	26 (7.2%)	162 (45.1%)	160 (44.6%)
Family problems such as alcoholism or marital disorder often contribute to the child’s ADHD.	4 (1.1%)	26 (7.2%)	78 (21.7%)	134 (37.3%)	117 (32.6%)
ADHD can be the result of the child not trying hard enough to control his/her behavior.	3 (0.8%)	13 (3.6%)	69 (19.2%)	161 (44.8%)	113 (31.5%)
A child with ADHD should be treated if a doctor recommended it.	1 (0.3%)	11 (3.1%)	31 (8.6%)	112 (31.2%)	204 (56.8%)
I would be reluctant to learn specialized teaching techniques to treat a child’s ADHD.	47 (13.1%)	96 (26.7%)	86 (24%)	78 (21.7%)	52 (14.5%)
Social skills training can be helpful for children with ADHD.	9 (2.5%)	1 (0.3%)	36 (10%)	173 (48.2%)	140 (39%)
Clear, consistent rules and consequences are helpful in treating children with ADHD.	3 (0.8%)	4 (1.1%)	36 (10%)	166 (46.2%)	150 (41.8%)

The relationship between knowledge, attitude toward ADHD, and demographic data among female primary schoolteachers is demonstrated in Table 4. The level of knowledge was significantly higher among private schoolteachers and freshly graduated (p=0.011 and 0.002, respectively). Furthermore, schoolteachers teaching second grade and specializing in learning difficulties had higher knowledge (p=0.019 and 0.016, respectively). Finally, the median knowledge score was significantly higher among schoolteachers who never heard of or attended any course/training about ADHD, those who gained their knowledge from scientific courses and during study years, and whoever taught ADHD children (p=0.020, 0.000, 0.016, 0.003 respectively). Unlikely, public schoolteachers, those with higher age and years of experience, holding a post-graduate degree, whoever asked to assess a child with ADHD or have ADHD child showed more significantly favorable attitudes (p=0.009, 0.045, 0.000, 0.001, 0.047 and 0.034 respectively). On the other hand, there was no significant association between knowledge, attitude level, and other demographic characteristics of female primary schoolteachers in Taif City, Saudi Arabia (Table 4).

**Table 4:** Factors associated with knowledge and attitude of female primary schoolteachers toward ADHD in Taif City, Saudi Arabia (N =359) * significant at p < 0.05, significant findings are shown in bold P1: p-value for ADHD-related knowledge, P2: p-value for ADHD attitude a: Mann-Whitney U test, b: Kruskal-Wallis test IQR: Inter-quartile range

Variable	Knowledge median (IQR)	Attitude median (IQR)	p-value
School type			P1=0.011* ^a^ P2=0.009* ^a^
Public	12(0-25)	49(12-60)	
Private	15(0-24)	47(34-58)	
Age			P1=0.117 ^b^ P2=0.045* ^b^
24-33	15(0-24)	48(35-60)	
34-43	12(0-22)	47 (12-60)	
44-53	13(0-25)	49(31-60)	
54-63	13(2-22)	50(39-60)	
Education			P1=0.868 ^b^ P2=0.001* ^b^
Secondary and lower	13(0-22)	50 (31-60)	
University bachelor's degree	13(0-25)	48(12-60)	
Postgraduate degree	12(4-22)	53(42-60)	
Marital status			P1=0.384 ^b^ P2=0.249 ^b^
Single	14(1-24)	48 (12-60)	
Married	12(0-25)	48(31-60)	
Divorced	14(0-23)	48(35-60)	
Widowed	13(7-21)	50 (45-60)	
Years of experience			P1=0.002^ b^ P2=0.000 ^b^
Equal or less than 5	15(0-24)	48 (35-60)	
6-15	12(0-24)	47(12-60)	
16-25	11(0-20)	49(36-60)	
Equal or more than 26	14(0-25)	50(39-60)	
Teaching grade #			
1^st^ grade			P1=0.175 ^a^ P2=0.668 ^a^
Yes	14(0-22)	48(31-60)	
No	12(0-25)	48(12-60)	
2^nd^ grade			P1=0.019 ^a^ P2=0.432 ^a^
Yes	14(0-25)	48(31-60)	
No	12(0-23)	49(12-60)	
3^rd^ grade			P1=0.478 ^a^ P2=0.262 ^a^
Yes	13(0-25)	48(31-60)	
No	13(0-23)	49(12-60)	
4th grade			P1=0.269 ^a^ P2=0.0194 ^a^
Yes	12(0-24)	48(12-60)	
No	14(0-25)	49(31-60)	
5^th^ grade			P1=0.554 ^a^ P2=0.195 ^a^
Yes	12(0-25)	48(12-60)	
No	14(0-24)	49()31-60	
6^th^ grade			P1=0.435 ^a^ P2=0.344 ^a^
Yes	12(0-25)	49(12-60)	
No	14(0-24)	48(31-60)	
Teaching specialty #			P1=0.016*^ b^ P2=0.034 ^b^
Languages	13(0-22)	49(36-60)	
Mathematics	12(0-25)	48(12-60)	
Science	12(0-21)	49(36-58)	
Education	14(0-23)	49(36-60)	
Kindergarten	10(0-19)	45(41-54)	
Learning difficulties	18(7-25)	44(38-54)	
General	13(1-22)	49(39-60)	
Digital education	14(0-21)	46(35-58)	
Physical education	15(0-21)	48(39-54)	
Life skills	10.5(0-22)	48(36-60)	
Have you ever heard about ADHD?			P1=0.020*^ a^ P2=0.281^ a^
No	10(0-17)	46(36-60)	
Yes	13(0-25)	48 (12-60)	
Have you ever attended any courses/training about ADHD?			P1=0.000*^ a^ P2=0.648^ a^
Yes	17.5(2-25)	48 (37-60)	
No	12(0-23)	48(12-60)	
During the years of teaching, do you think you have enough information about ADHD?			P1=0.000*^b^ P2=0.080^ b^
No	10(0-21)	48(12-60)	
Yes	17(0-25)	50(37-60)	
Not sure	13(0-24)	49(34-60)	
Have you ever been asked to assess a child suspected to have ADHD?			P1=0.000*^ a^ P2=0.047*^ a^
Yes	15(0-25)	49(34-60)	
No	11(0-23)	48(12-60)	
Have you ever taught a child who has ADHD?			P1=0.003*^ a^ P2=0.933^ a^
Yes	14(0-25)	48(34-60)	
No	11(0-23)	48(12-60)	
Number of children You taught with ADHD in years			P1=0.000*^b^ P2=0.080^ b^
1.00	14(1-24)	48(34-60)	
2.00	14(0-21)	48(33-60)	
3.00	15(4-19)	49(42-59)	
4.00	15(0-25)	50(38-60)	
The main source of information about ADHD (n=338)			P1=0.016*^ b^ P2=0.034^b^
Specialized books or magazines	17(0-24)	48(35-60)	
Websites or scientific articles	13(0-24)	49(34-60)	
Television/media	12(0-21)	48(12-60)	
Social media	12(0-22)	48(36-60)	
Scientific courses	21(14-25)	42(38-50)	
during the study years	21(10-22)	48(48-51)	
during the work	15(4-18)	44(38-47)	
I have an ADHD child	16(2-22)	50(43-60)	

Table 5 illustrates Spearman's rank correlation matrix of the bivariate variables in relation to knowledge and attitude toward ADHD. The knowledge general subscale, knowledge symptoms and diagnosis subscale, and knowledge treatment subscale had a positive, strong correlation with the total knowledge score (rs=0.883, rs=0.837, and rs=0.871, p=0.000, respectively). In addition, the attitude score had a significant positive weak correlation with the total knowledge score, knowledge general subscale, knowledge symptoms and diagnosis subscale, and knowledge treatment subscale (rs=0.210, rs=0.232, rs=0.225 p=0.000, and rs=0.115 p=0.028, respectively).

**Table 5:** Spearman's rank correlation matrix of the bivariate variables in relation to knowledge and attitude toward ADHD ** correlation is significant at the 0.01 level (2-tailed), * correlation is significant at the 0.05 level (two-tailed), # p-value is illustrated in italic format rs: spearman correlation coefficient

		Total knowledge score	Attitude score	Knowledge general subscale	Knowledge symptoms subscale	Knowledge treatment subscale
Total knowledge score	r_s_	1.000	0.210**	0.883**	0.837**	0.871**
	p	.	0.000	0.000	0.000	0.000
Attitude score	r_s_	0.210**	1.000	0.232**	0.224**	0.115*
	p	0.000	.	0.000	0.000	0.028
Knowledge general subscale	r_s_	0.883**	0.232**	1.000	0.628**	0.653**
	p	0.000	0.000	.	0.000	0.000
Knowledge symptoms and diagnosis subscale	r_s_	0.837**	0.225**	0.628**	1.000	0.641**
	p	0.000	0.000	0.000	.	0.000
Knowledge treatment subscale	r_s_	0.871**	0.115*	0.653**	0.641**	1.000
	p	0.000	0.028	0.000	0.000	.

The ADHD knowledge, attitude, and demographic data among female primary schoolteachers in Taif City, Saudi Arabia, were illustrated using adjusted ORs and their confidence intervals in (Table 6). Logistic regression revealed that participants who ever taught a child who has ADHD (OR = 0.054, 95% CI = 0.003, 0.973, p = 0.048) revealed that teachers’ who never taught an ADHD child had a reduction by 94.6 % in the ADHD knowledge compared to teachers’ whoever teach an ADHD child. On the other hand, any increase in the number of ADHD children teachers teaches will significantly increase teachers’ knowledge (OR = 1.789, 95% CI = 1.094, 2.923, p = 0.020). Lastly, schoolteachers specialized in learning difficulties show significantly higher knowledge scores than those with other specialties (OR = 1056, 95% CI = 2.471, 45197, p = 0.024) (Table 6).

**Table 6:** Regression analysis of ADHD knowledge according to significant variables among female primary schoolteachers in Taif City, Saudi Arabi CI: confidence interval, df: degrees of freedom # logistic regression: outcome: ADHD knowledge (chi-square X2= 69.514, p < 0.000**) # predictors: school type (reference: governmental), Have you ever heard about ADHD? (reference: no), ADHD source of information (reference: no), Have you ever attended any course/training about ADHD? (reference: no), During the years of teaching, do you think you have enough information about ADHD? (reference: no), Have you ever been asked to assess a child suspected to have ADHD? (reference: no), Have you ever taught a child who has ADHD? (reference: no), Number of children you taught with ADHD, specialty (reference: language), attitude score, grade 2 (reference: no)

	Wald	df	Sig.	Odds ratio (95% CI)
School type	0.869	1	0.351	0.225 (0.01, 5.16)
Years of teaching experience	1.396	1	0.237	0.912 (0.78, 1.06)
Have you ever heard about ADHD?	0.000	1	0.998	761155 (0.00, .)
ADHD source of information	7.689	8	0.464	
Specialized books or magazines	0.000	1	0.998	5132635 (0.00, .)
Websites or scientific articles	0.000	1	0.998	1945396 (0.00, .)
Television/media	0.000	1	1.000	0.588 (0.00, .)
Social media	0.000	1	0.998	1251152 (0.00, .)
Scientific courses	0.000	1	0.998	8172430 (0.00, .)
during the study years	0.000	1	0.997	2896424 (0.00, .)
during the work	0.000	1	1.000	1.115 (0.00, .)
I have an ADHD child	0.000	1	0.997	2786525 (0.00, .)
Have you ever attended any courses/training about ADHD?	0.520	1	0.471	0.244 (0.05, 11.32)
During the years of teaching, do you think you have enough information about ADHD?	0.000	1	0.994	103008967 (0.00, .)
Have you ever been asked to assess a child suspected to have ADHD? (1)	0.196	1	0.658	1.841 (0.12, 27.4)
Have you ever taught a child who has ADHD?	3.913	1	0.048*	0.054 (0.00, 0.97)
Number of children you taught with ADHD	5.383	1	0.020*	1.789 (1.09, 2.92)
Specialty	6.253	9	0.714	
Mathematics	1.438	1	0.230	56.724 (0.07, 41710)
Science	0.000	1	0.998	0.000 (0.00, .)
Education	2.031	1	0.154	60.532 (0.21, 17096)
Kindergarten	0.000	1	0.999	0.000 (0.00, .)
Learning difficulties	5.075	1	0.024*	1056.8 (2.47, 451973)
General	2.559	1	0.110	364.34 (0.26, 500973)
digital education	0.000	1	0.999	0.000 (0.00, .)
Physical education	0.000	1	0.999	0.000 (0.00, .)
Life skills	1.007	1	0.316	17.092 (0.06, 4369)
ADHD attitude score	1.031	1	0.310	0.895 (0.72, 1.10)
Grade 2	1.215	1	0.270	3.644 (0.36, 36.34)
Constant	0.000	1	0.995	0.000

## Discussion

In this research, we explored ADHD knowledge and attitude of female primary schoolteachers in Taif City, Saudi Arabia, and studied factors that influence ADHD knowledge among Taif female schoolteachers. Results showed that more than 96% have poor ADHD knowledge, especially general and treatment ADHD knowledge. Saudi studies in Makkah, Jeddah, and Abha cities (Makkah 2013: 17.3%, Makkah 2014: 55.4%, Jeddah 2019: 24.5%, and Abha 2020: 16-22%) [22,19,25,26] and other international studies [16,18] showed low rates of good knowledge of ADHD among schoolteachers. These differences may be accounted for in the timing of data collection, differences in school settings, differences in the population age, years of experience, different ADHD knowledge assessment tools, and history of higher rates of having children diagnosed with ADHD. This finding was in contrast with another Saudi study carried out in Riyadh that reported a high overall rate of good knowledge (72%). More than half of the participating teachers in the Riyadh study had prior experience managing ADHD students, which may account for their high level of knowledge [23]. Although the teachers in the current study had a higher level of knowledge of the diagnosis and symptoms of ADHD, they had less awareness about the nature, causes, consequences, and treatment. Two-thirds of the teachers were aware that ADHD children are more noticeable in a classroom setting than in an unrestricted play situation, and they were also aware of one of the hallmark signs of ADHD which is that the children often fidget in their seats. However, the majority of teachers were unaware of the genetic role of ADHD, incorrectly believed that children with ADHD have an inflated sense of self-esteem, were unaware of the personalities of ADHD children, and thought that effective treatment for ADHD should be multifaceted and comprehensive. Teachers showed a positive ADHD attitude, which was in agreement with a study conducted in Pakistan (92.2%) [27] and contrary to previous studies reporting a low attitude toward this issue [28-31]. The observed disagreement between studies may result from disparities in sample size, sampling technique, study design, tool difference, participants, and cultural characteristics too. Moreover, teachers strongly agree that training is important in the behavioral management of ADHD but also hold a misperception about the effectiveness of the diet on the symptoms of ADHD as they thought incorrectly that the symptoms of ADHD will decrease with the reduction of sugar and or food additives intake. These findings are consistent with the findings of other studies [16,19,32], which showed that symptoms and diagnostic scores were much higher than treatment and general information scores. The poor teacher’s knowledge may contribute to delayed detection of ADHD and improper management of the associated children learning and behavioral problems.

Having recently graduated, being younger, or gaining knowledge during the study years had a beneficial impact on the level of teachers' knowledge of ADHD in the current study. This might be a result of the newly strengthened curricula that place an emphasis on ADHD. Moreover, schoolteachers who had never heard of or attended training on ADHD scored significantly higher on the knowledge scale; attending a course or training on ADHD is a form of professional development that can help teachers develop specialized knowledge and skills related to working with students with ADHD. Information about effective classroom strategies, behavior management approaches, and accommodations that can assist students with ADHD to succeed in school may be included in this professional development training. Alshehri et al. (2020) reported that teachers’ ADHD knowledge was significantly improved immediately after the teachers' training [26]. These findings are in line with those reported in earlier studies conducted in Saudi Arabia, which found that schoolteachers who are younger or received educational programs on ADHD had higher knowledge of ADHD [23,26,30,33]. However, Dessie et al. reported that having postgraduate studies and gaining ADHD knowledge from Internet searches helped to acquire knowledge of different issues of ADHD [24]. Although the majority of the population has Internet access, and it is a rapid and cost-effective method to get different information, it is possible that this information about the disease may be inaccurate or unreliable. Knowledge of the teachers in different studies is not the same [32,33].

The current study highlighted the relationship between teachers' high knowledge of ADHD and their specialization in learning difficulties or previous experience working with a child with ADHD. Importantly, the regression analysis revealed that teachers who had never taught an ADHD child had a reduction of 94.6% less ADHD knowledge than those who had. These findings highlighted the importance of providing teachers with training and support to improve their understanding of ADHD even if they had not yet taught a child with the disorder. This may explain why schoolteachers specializing in learning difficulties have significantly higher knowledge scores than those with other specializations. This is concordant with Al‑Moghamsi et al. [20] who found that special needs teachers had the highest score of knowledge regarding ADHD symptoms, and they had the highest score of knowledge of ADHD treatment, and teachers who reported previous experience with a child with ADHD expressed a higher level of overall knowledge. This was also reported in previous studies [34,35]. In contrast, another study conducted in Jeddah found that having a child diagnosed with ADHD or previously working in a special education school had no significant influence on knowledge scores [25]. The challenges of teaching children with ADHD may have prompted teachers to seek additional training information and resources. Finally, working in private schools may be influential, where private schoolteachers tended to have more comprehensive levels of knowledge of ADHD. This is somewhat consistent with Kern et al. [36]. Contrary to this finding, Hosseinnia et al. determined that participants teaching in public schools had better knowledge and attitude [30], while Badleh [37] did not discover a statistically significant correlation between knowledge and attitude and the school type. The possible reason for such a result could be that teachers in private schools had to attend more diverse training courses than teachers in public schools due to the intense competition between teachers while applying for vacancies in private schools.

Regarding factors associated with the participant’s attitude toward ADHD; public school instructors who are older, have more years of experience, and have a post-graduate degree are more likely to have positive attitudes toward students with ADHD. Plausible explanations for such findings could be attributed to co-occurrences of higher years of experience and the likelihood of working with a larger number of students with ADHD and the accumulation of information through either experience or education [38]. This experience may have helped them develop a better understanding of the condition, as well as effective strategies for working with students with ADHD. A positive relationship was found between knowledge and attitude. This indicates that those who know more about ADHD also have a more tolerant attitude. Probably the key to developing tolerant attitudes would then be increasing teachers’ knowledge. This increased understanding and experience may lead to more positive attitudes toward students with ADHD. This finding was consistent with Tyagi et al. [39], while, contrary to this finding, Hosseinnia et al. determined a significant inverse relationship between the means of knowledge and attitude of teachers and their age. This shows that special courses or lectures or formal instructions on ADHD students should be provided [30].

There are a number of strengths of this study, including the inclusion of an appropriate sample size, enrollment of a random sample, inclusion of private and public schools from different taif districts, and the use of a reliable and valid tool. Furthermore, this is the first study in Taif to examine teachers' ADHD knowledge. However, there are also some limitations to consider. Firstly, the collection technique was self-reported data, which may involve recall bias. Secondly, data were based on a cross-sectional and causation cannot be assured. Finally, the study included only female teachers because the Saudi Ministry of Education issued a Cabinet Resolution about the early childhood initiative aimed to enhance enrolment and performance levels in the primary classes by assigning only female teachers to teach young male and female pupils starting in 2019. Interventional studies are recommended to assess the change in schoolteachers' ADHD knowledge after training.

## Conclusions

In conclusion, this study highlights three major important issues for primary school children's health in Taif. First, teachers’ scores on KADDS were very low compared to previous research conducted in other Saudi cities, indicating a serious knowledge gap on ADHD. Second, teachers' prior experience with ADHD, training, and specialization on learning difficulties were all positively associated with their level of knowledge of ADHD. Third, teachers' attitude toward ADHD is correlated positively with their knowledge.
